# RFID-Based Microwave Biosensor for Non-Contact Detection of Glucose Solution

**DOI:** 10.3390/bios11120480

**Published:** 2021-11-26

**Authors:** Minjia Gao, Tian Qiang, Yangchuan Ma, Junge Liang, Yanfeng Jiang

**Affiliations:** Department of Electronic Engineering, School of Internet of Things Engineering, Jiangnan University, Wuxi 214122, China; 6201924078@stu.jiangnan.edu.cn (M.G.); 6201924123@stu.jiangnan.edu.cn (Y.M.); jgliang@jiangnan.edu.cn (J.L.); yanfeng_jiang@yahoo.com (Y.J.)

**Keywords:** RFID, non-contact measurement, microwave biosensor, complementary split-ring resonator, glucose concentration

## Abstract

Due to the increasing number of diabetic patients, early monitoring of glucose levels is particularly important; therefore, glucose biosensors have attracted enormous attention from researchers. In this paper, we propose a glucose microwave biosensor based on RFID and achieve a non-contact measurement of the concentration of glucose solutions. The Reader is a complementary split-ring resonator (CSRR), and the Tag is comprised of a squared spiral capacitor (SSC). A polydimethylsiloxane microfluidic quantitative cavity with a volume of 1.56 μL is integrated on the Tag to ensure that the glucose solution can be accurately set to the sensitive area and fully contacted with the electromagnetic flux. Because the SSC exhibits different capacitances when it contacts glucose solutions of different concentrations, changing the resonant frequency of the CSRR, we can use the relationship to characterize the biosensing response. Measurement results show that bare CSRR and RFID-based biosensors have achieved sensitivities of 0.31 MHz/mg·dL^−1^ and 10.27 kHz/mg·dL^−1^, and detection limits of 13.79 mg/dL and 1.19 mg/dL, respectively, and both realize a response time of less than 1 s. Linear regression analysis of the abovementioned biosensors showed an excellent linear relationship. The proposed design provides a feasible solution for microwave biosensors aiming for the non-contact measurement of glucose concentration.

## 1. Introduction

Diabetes is a chronic metabolic disease characterized by elevated blood sugar levels, which can cause severe damage to body organs such as the heart, blood vessels, eyes, kidneys, and nerves over time [1,2]. Therefore, the early detection and screening of diabetic patients are very important. It is crucial to develop a method to realize early detection of glucose for the treatment and management of diabetes [3].

Nowadays, biosensing detection conversion plays a very important role in the sensing system because of several key factors such as portability, accuracy, and low cost [4]. Compared with traditional glucose detection methods, such as fluorescent and colorimetric [5,6], the latest biosensors convert glucose solutions of different concentrations into corresponding output electrical signals with higher accuracy, better linearity, as well as quicker response times [7,8]. Since the test solution usually exhibits different conductivity, refractive index, and dielectric constant under different concentration conditions, biosensors are usually categorized into electrochemical biosensors, optical biosensors, and microwave biosensors [9,10,11]. Electrochemical biosensors are widely accepted for the detection of glucose solutions due to their excellent selectivity, high sensitivity, simple operation, and effective cost. The operating mechanism is to detect biomarkers by adding specific enzymes to the electrodes [7,12,13]. However, the introduction of foreign media, such as some small molecules that serve as channels between enzymes and motor electrodes, slows the response, reduces the performance, and ultimately leads to deteriorating reliability. Moreover, another key factor limiting the application of electrochemical sensors is that the electrolytes need to be replenished regularly, which increases subsequent costs. With regard to optical biosensors, non-contact and non-destructive measurement methods can be realized based on optical principles and have excellent color recognition performance for the test solution added with fluorescent markers [14,15,16]. However, due to the complex measurement system of optical devices, long calibration and stabilization time, and the detection deviation caused by the influence of ambient light, it cannot be popularized on a large scale. Recently, microwave biosensors, which are considered to be a promising and competitive candidate for the realization of the third-generation glucose biosensor, have attracted great attention [17,18,19]. Microwave biosensors rely on unique advantages such as having lower performance degradation during service time when compared with other types of biosensors, are not sensitive to environmental conditions (such as external light, noise interference), and can maintain stability in long-term complex environments [20]. Microwave biosensors can perform rapid detection of biomarkers in real time without the need for pre-stabilization time. More importantly, microwave biosensors can realize a non-contact detection of solution concentration [21].

In this work, we propose a biosensor based on the concept of RFID, which consists of three parts, namely the Reader, Tag, and PDMS-based microchannel. Benefiting from the novel biosensor design with complementary split-ring resonator (CSRR) and squared spiral capacitor (SSC) structure, it was found that the glucose concentration is proportional to the resonant frequency of the biosensor, which implies that the resonant frequency can be applied to map the different glucose levels, indicating the feasibility of the proposed biosensor in glucose detection. Moreover, the sensing Tag was made on a glass substrate, which is a biologically compatible substrate and is quite promising in biological fields. The microfluidic channel based on PDMS can simulate biological structures and even biological tissues. Furthermore, the volume of test solution required was very low (1.56 μL). The Reader could receive microwave signals from Tag; therefore, the purpose of non-contact detection and monitoring of blood glucose concentration can be achieved. Finally, the proposed biosensor based on RFID with a fast response time, robust and accurate test results, and excellent non-contact performance were realized, which opens up new routes for many applications such as the non-contact detection of glucose concentration.

## 2. Materials and Methods

### 2.1. Biosensor Design

**Figure 1:**
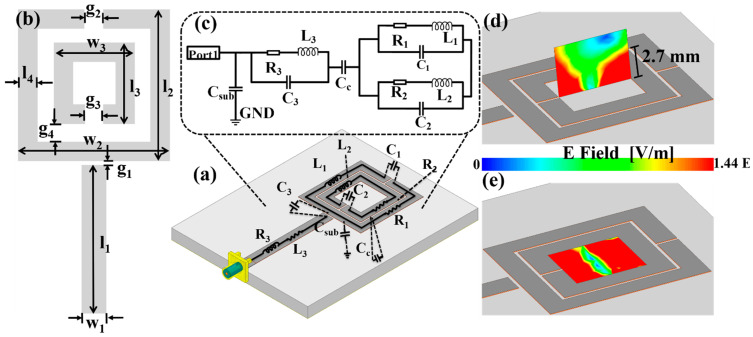
(**a**) Structure and size of the proposed Reader, (**b**) the equivalent circuit of the proposed split-ring structure, (**c**) the proposed Reader structure model, (**d**) the electric field intensity distribution on the vertical, and (**e**) horizontal planes in the middle area of the Reader.

An RFID-based microwave biosensor with microchannels was designed and simulated using a high-frequency structure simulator. The simulated Reader structure, along with its lumped circuit model, is presented in Figure 1a. The Reader is a CSRR structure with a high *Q*-factor, which is used for accumulating and transferring the energy to the Tag. Electromagnetic resonators demonstrated for the sensing of liquid samples are usually designed in the microwave regime. Geometric sizes associated with this frequency regime are easy to handle and therefore suitable for portable applications. Going further down in size and operating at mm wavelength and THz frequencies has the drawback of reduced reliability since, at high frequencies, the effective ε_r_ of the liquid samples will be changed due to increased absorption.

The Reader was fabricated on a printed circuit board substrate with a dielectric constant of 9.6, loss tangent of 0.003, and a thickness of 1.524 mm. The CSRR structure was made of 35 μm copper. In order to prevent the copper from oxidation and to make it convenient for subsequent soldering, a surface tin sinking process was used. The structure and size of the proposed structure is shown in Figure 1b, in which l_1_ = 11.3 mm, w_1_ = 1.5 mm, l_2_ = w_2_ = 9.2 mm, l_3_ = w_3_ = 6.4 mm, g_2_ = g_3_ = g_4_ = 0.2 mm, and l_4_ = 1.2 mm. Figure 1c shows the equivalent circuit of CSRR, which is a conductive circuit path with three gaps on it. The electrical behavior of the CSRR can be modeled with an equivalent resistor *R*, capacitor *C*, and inductor *L*. The gap capacitance *C_g_*, substrate capacitance *C_sub_*, and coupling capacitance *C_c_* are shown in the below equation,

(1) $${C=C}_{g}{+C}_{sub}{+C}_{c}$$

The *RLC* circuit model parameters are inherent parameters of CSRR geometry, which is defined by gap width (g_1_, g_2_, g_3_), conductive path width (w_1_, w_2_, w_3_), the distance between two conductive paths (g_4_), and the length of conductor (l_1_, l_2_, l_3_). Analytical models are developed in this work linking the *RLC* quantities to the geometry parameters of the CSRR. Following these models, the resonant frequency *f*_0_ and *Q* factor of the CSRR can be derived based on the below equations [22]:

(2) $$f_{0}=\frac{1}{2\pi \sqrt{{L(C}_{g}{+C}_{sub}{+C)}_{c}}}$$

(3) $$Q=\frac{1}{R}\sqrt{\frac{L}{C_{g}{+C}_{sub}{+C}_{c}}}$$

Compared with *C_g_*, *C_sub_* is too small and is ignored; *C_c_* is a constant. Therefore, although a higher *C_g_* can potentially concentrate the electric field in a single region of the CSRR and increase the sensitivity, based on Equation (3), a high total *C* can cause a deterioration in *Q*, which presents a design trade-off. The nominal *Q* of the Reader simulated, as such, can achieve a value of 46. Moreover, *Q* is observed to decrease with further dielectric loading, especially in the context of sensing with liquid samples. Therefore, in order to maintain the high *Q* of the biosensor, a CSRR structure with optimized key dimensions and good performance was adopted, which is illustrated in Figure 1d,e. It is obvious to see from the schematic diagram that there is an electric field distribution in the middle area of the Reader whether it is vertical or horizontal, which also proves that this area is sensitive and can radiate the signal upwards about 2.7 mm, and such a radiation distance is enough for energy transmission.

**Figure 2:**
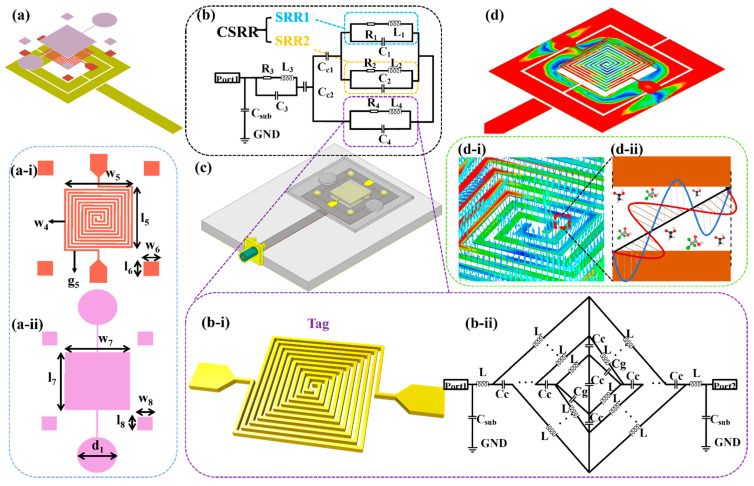
(**a**) Proposed 3D structure of the biosensor and the structure and size of (**a-i**) Tag and (**a-ii**) microchannel, (**b**) the equivalent circuit model of the biosensor, and (**b-i**) Tag structure and its (**b-ii**) equivalent circuit model. (**c**) The model of the proposed biosensor, (**d**) the electric field distribution of Reader and Tag at resonant frequency, and (**d-i**) Tag’s electric field coupling model including (**d-ii**) the schematic diagram of the electromagnetic wave acting inside the metal wire.

The 3D structure of the RFID-based biosensor is illustrated in Figure 2a. The microchannel, Tag, and Reader are located from top to bottom. The design of the Reader has been explained above. The specific structure of the designed Tag is shown in Figure 2(a-i). The specific dimensions are described as follows, l_5_ = 3.9 mm, w_5_ = 4 mm, g_5_ = w_4_ = 0.1 mm, and l_6_ = w_6_ = 0.9 mm. The Tag was designed with two metal wires meandered into a squared spiral structure. Four square metal blocks were prepared around the SSC. This is to align with the designed microchannel, which is depicted in Figure 2(a-ii), and the dimensions are l_7_ = 4.3 mm, w_7_ = 4.2 mm, l_8_ = w_8_ = 1 mm, d_1_ = 2.6 mm, respectively. Its middle structure can just cover the area of the Tag. Four calibration modules were slightly larger than the metal block of the Tag in order to facilitate subsequent alignment and bonding. The Tag was fabricated through a Ti/Au (20/80 nm) sputtering process followed by a Cu (5 μm) plating process and made on a 0.8 mm glass substrate (SCHOTT B 270). The usage of a glass substrate can not only lower the cost but also reduce the dielectric loss of the substrate [22]. The microchannel was made of PDMS because of its good biological properties and metal compatibility. The PDMS microchannel was attached to the glass substrate by plasma treatment and hotplate heating, and the 3D simulation diagram is shown in Figure 2c when the RFID chip is placed in the middle of the Reader. Figure 2b displays the biosensor equivalent circuit, the specific structure of the Tag is shown in Figure 2(b-i), and the corresponding equivalent circuit model is represented in Figure 2(b-ii). As depicted in Figure 2d, the electromagnetic signal passes through the Reader, and the energy can pass through the glass substrate and radiate to the Tag. The electromagnetic signal propagates between the metal lines of the Tag, and its direction will change with the change of the phase. At the resonant frequency of 3.77 GHz, the direction of the electric field lines at a certain phase is shown in Figure 2(d-i). If there is a biological medium between the metal wires, the electromagnetic signals will interact with them, as shown in Figure 2(d-ii).

The electromagnetic wave radiated by the Reader strongly couples with the SSC Tag, and the simulated current density of the biosensor at the resonant frequency is illustrated in Figure 2d. Therefore, the SSC Tag is able to transform a small change in the permittivity caused by the changing of glucose concentration into effective capacitance variations. Accordingly, the CSRR transforms the shift in resonant frequency. The capacitance of SSC is based on the finger width (*WF*), finger length (*LF*), finger gap (*GF*), the gap between electrode and feed line, and feed line width. The 3D schematic, dimension marker, and equivalent circuit of the proposed SSC structure are shown in Figure 2(b-i, a-i, b-ii). The equivalent circuit of the SSC is represented with series/parallel combinations of inductor and capacitor according to design conductive path, coupling, and gap capacitance, respectively. In the presented equivalent structure, *L* represents inductance, *C_g_* represents gap capacitance, and *C_c_* represents coupling capacitance. As for conventional SSC models, *C_g_* is considered as an essential factor in deciding overall device capacitance. However, after the advancement of micro-fabrication techniques, *C_c_* becomes a significant parameter for capacitive variation. The overall circuit capacitance of SSC can also be represented based on an analytical semiempirical equation as [23,24],

(4) $$C_{SSC}=\left[\varepsilon_{o}\varepsilon_{sub}\left(\frac{{1+\varepsilon }_{s}}{2}\right)\times \frac{K\left(\sqrt{1-K^{2}}\right)}{K(k)}{+\varepsilon }_{o}\varepsilon_{s}\frac{t}{a}+{\left\{\frac{K\left(k\right)}{\varepsilon_{o}\varepsilon_{s}K\left(\sqrt{1-k^{2}}\right)}\right\}}^{-1}\right]\times LF$$

(5) $$K=\left(1+\frac{2\times WF}{2\times GF+WF}\right)\times \left(\sqrt{\frac{1}{1+\frac{2\times WF}{GF}}}\right)$$

where *ε_o_* represents the effective permittivity of free space (*ε_o_* = 8.854 × 10^−12^ F/m), *ε_sub_* represents substrate permittivity, and *ε_s_* is the permittivity of the glucose sample. *K* represents the first elliptical integral, *k* represents the ratio of the finger width and finger gap (*WF*/*GF*), and *LF* represents the length of the coupled meandered line. Equation (4) represents the device capacitance and is directly proportional to the complex permittivity of testing materials. In a testing experiment, the complex permittivity of the tested solution will change with the change of the solution concentration that correspondingly alters the overall device capacitance.

Since glucose solutions with different shapes, volumes, and test positions could lead to a detection accuracy issue, PDMS microchannel was applied to achieve the shape setting (fixed surface area and thickness), volume setting, and test position setting of the tested sample for a quantitative measurement with only 1.56 μL (4.0 mm × 3.9 mm × 0.1 mm), which can eliminate the influence caused by shape, volume, and test position of the glucose sample during the test, ensuring the accuracy of measurement results.

### 2.2. Biosensor Operating Mechanism

According to the Debye dispersion model, a more general complex quantity expression for the glucose sample can be expressed using the dielectric constant ($\varepsilon_{s}^{\prime }$) and loss factor ($\varepsilon_{s}^{\prime \prime }$) as follows [24],

(6) $$\varepsilon_{s}{=\varepsilon }_{s}^{\prime }+j\varepsilon_{s}^{\prime \prime }=\left[\frac{{(\varepsilon }_{sub}{-\varepsilon }_{\infty })}{{1+\omega }^{2}\tau^{2}}{+\varepsilon }_{\infty }\right]+j\left[\frac{{(\varepsilon }_{sub}{-\varepsilon }_{\infty })\omega \tau }{{1+\omega }^{2}\tau^{2}}\right]$$

The above equation is an analytical semiempirical equation, which was used to study the influence of the sample glucose solution on permittivity. Due to the compositional characteristics of the molecule, when glucose anhydrase is dissolved in water, the monosaccharide molecules (C_6_H_12_O_6_) contain a higher number of -OH groups compared with the -H bands, which leads to less available water to interact with the alternating current (AC) field. This explains why the dielectric constant of the water-glucose solution was lower than water. Furthermore, the molecular weight of glucose is as high as 180.2 kDa, and it is ten times the molecular weight of water molecules. Thus, the glucose molecule is much heavier than the water molecule, which helps to explain the dielectric mechanism of the water-glucose solution. Firstly, the large size caused a more pronounced viscous effect, leading to a difficult rotation with the AC field. Secondly, a large dipole moment is unable to provide the molecule with enough compactness to facilitate reorientation with the AC field. According to Equation (6), the viscous effect is directly proportional to the concentration level of glucose. As a consequence, different glucose solutions lead to differences in their dielectric constants. The differences in dielectric constants correspondingly change the capacitance of the SSC Tag, and the change in the capacitance of SSC influences the capacitance of the overall RFID biosensor, which causes the reflection parameters of the CSRR resonator and ultimately reflects the deviation of the resonant frequency.

### 2.3. Microwave Detection Methods

**Figure 3:**
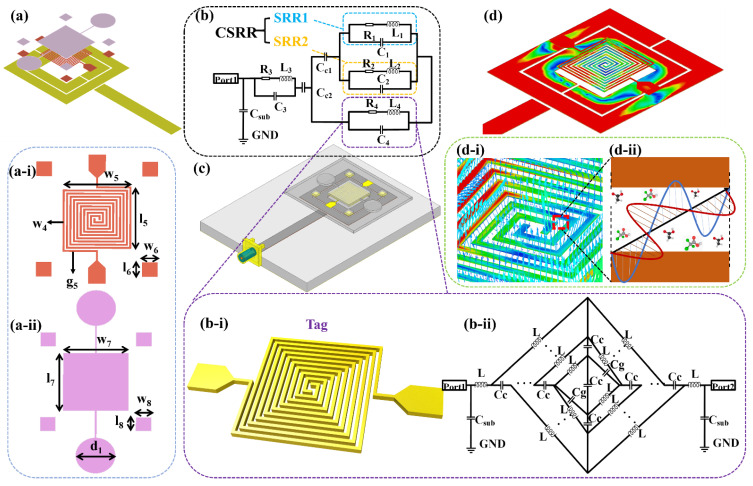
The fabricated RFID chip, including (**a**) Reader, (**b**) Tag, (**c**) RFID chip, and (**d**) microchannel.

The proposed biosensor was constructed from a CSRR Reader and an SSC Tag integrated with a PDMS microchannel. The change of Tag capacitance results in a resonant frequency shift of the Reader. To interrogate the SSC Tag without contact, the Reader was coupled with the Tag. Through monitoring the reflection coefficient S_11_ of the Reader, the measured biosensor response could be obtained. Before conducting the experiment, an SMA needed to be soldered to the Reader; the fabricated Reader is shown in Figure 3a. Before aligning and placing the Tag in the middle area of the Reader, the microchannel was fixed on the Tag. PDMS (Slygard 184, Dow Corning, USA) was mixed with 10% by weight of curing agent. The mixture was poured onto an SU-8 mold and was degassed in a vacuum chamber. After that, it was cured and solidified at 90 °C for 30 min on a hotplate. The formed PDMS microchannels were then cut and punctured to create the inlet/outlet holes; the structure and size can be observed from Figure 3d. After perforating the microchannel, it was aligned and bonded with the Tag on the glass substrate. The fabricated Tag with dimension marker on the glass substrate is shown in Figure 3b. Inserting the catheter into the previously punched hole and assembling the various parts together, the proposed biosensor was obtained, as shown in Figure 3c.

**Figure 4:**
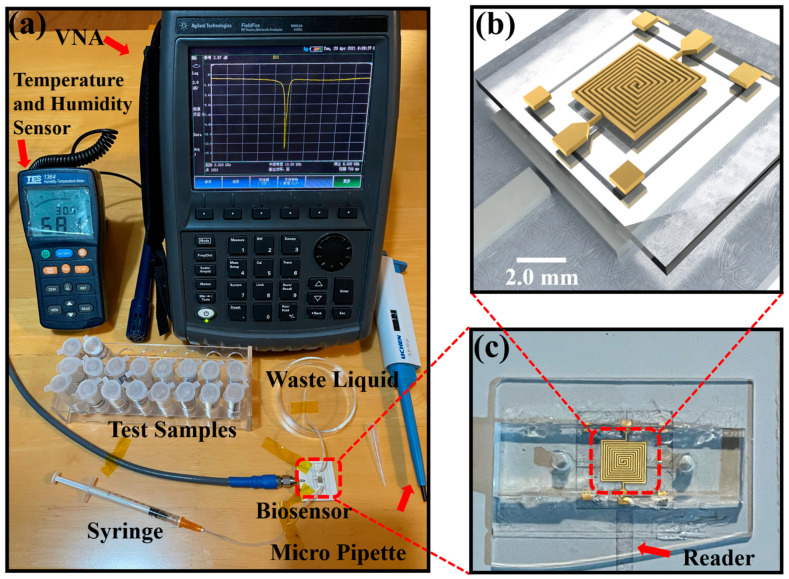
(**a**) Proposed experimental setup for chip measurement of detecting variable glucose level. (**b**) The 3D schematic diagram of RFID chip. (**c**) The fabricated biosensor.

In order to measure the microwave response of the proposed biosensor, a measurement platform was successfully constructed, consisting of a vector network analyzer (VNA), an under test biosensor, a quantitative micropipette, and a temperature and humidity sensor (Figure 4a). Ten different glucose samples were prepared, ranging from 25 to 600 mg/dL (25, 50, 100, 150, 200, 250, 300, 400, 500 and 600 mg/dL). These samples were composed of a mixture of glucose anhydrose and deionized (DI) water. The VNA was initially calibrated using a mechanical calibration kit with open, short, and load testing capabilities. After calibration, the VNA was set to 1 GHz bandwidth to measure the reflection coefficient (S_11_) over 1001 equally spaced data points between 3.5 GHz and 4.5 GHz. Before injecting the glucose solutions of different concentrations into the catheter through a syringe, we first tested and collected the relevant microwave parameters of the bare Reader and the bare chip for subsequent comparative analyses. All the experimental sample solutions were under the experimental conditions of temperature and humidity ranging from 29.2 °C to 30.6 °C and 66.9% RH to 68.4% RH, respectively. This was to reduce the influence of environmental factors on the final experimental results. The 3D schematic with dimension marker of the biosensor is shown in Figure 4b. Inside the red line is the designed biosensor, and the enlarged image of the biosensor structure is shown in Figure 4c.

## 3. Results and Discussion

### 3.1. Liquid Dielectric Loading

**Figure 5:**
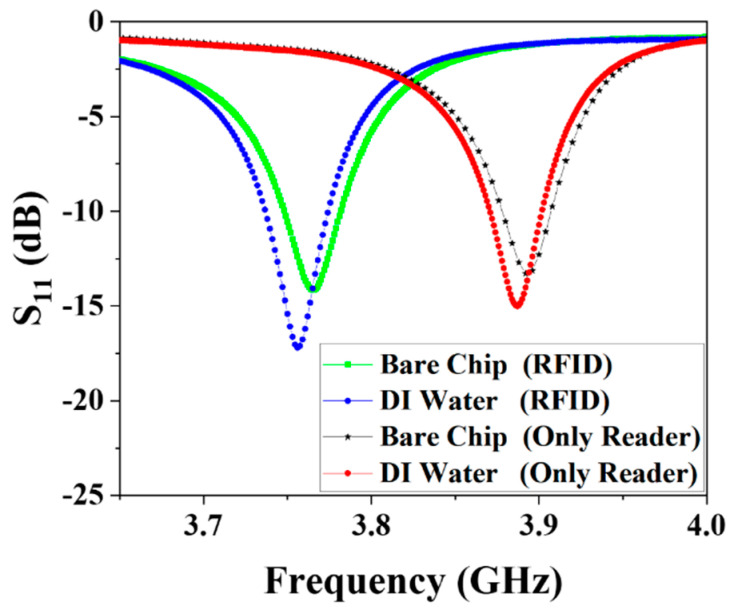
Measured reflection spectra (S_11_) for Reader and RFID-based biosensor. Microchannels are kept empty and filled with DI water, respectively, while simultaneous measurements are conducted.

Measured spectra for unloaded and DI water-loaded microchannels are shown in Figure 5. When the Tag is in the sensing area of the Reader, results demonstrate that the resonant frequency of the biosensor will shift approximately 150 MHz. This is because whether RFID is made of a glass substrate or metal, they all have a dielectric constant, which greatly enhances the capacitance of the Reader capacitor and causes a significant drop in the resonant frequency. The offset for the RFID biosensor occurs because the DI water is completely filled in the 10 μm metal line gap, the original air medium is replaced by the DI water medium, so the effective dielectric constant is changed, leading to a change in capacitance, which causes the resonant frequency shifting of the entire system. If the intertwined metal wire of the Tag is unrolled, the sensing capacitance of the Tag will increase compared to the sensing capacitance of the Reader, and when a larger sensing capacitance exists, the change in the dielectric constant will be more obvious. This is why the RFID-based biosensor in Figure 5 changes more when DI water is added to it than just to a bare Reader.

### 3.2. Glucose Solution Loading

**Figure 6:**
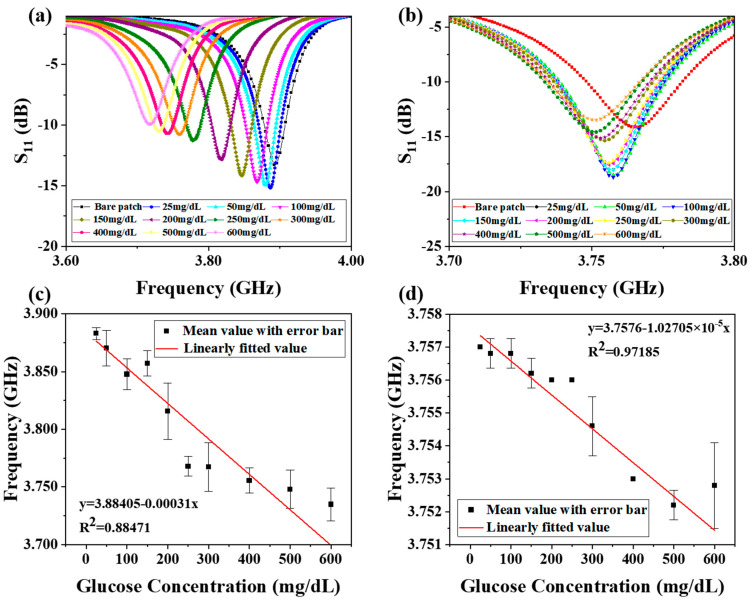
Measured S_11_ for different concentrations of glucose sample solutions (25–600 mg/dL): (**a**) bare Reader and (**b**) RFID system. Linearly fitted resonant frequency, including the mean value of resonant frequencies with error bars. (**c**) Reader structure (R^2^ = 0.88471, RSD < 1%) and (**d**) RFID system (R^2^ = 0.97185, RSD < 1%).

Glucose solutions with various concentrations (25–600 mg/dL) were introduced by syringe through the microchannels to measure the Reader and RFID separately. In the experiment, the temperature and humidity changes of 1.4 °C and 1.5% RH had a negligible influence on the dielectric constant (real part and imaginary part < 1) of the glucose solution. Therefore, the effect of temperature and humidity on the capacitance was almost negligible, so the change in the resonant frequency was almost negligible. The gap of the outside split-ring was selected to test the sensitivity of the Reader. This is because, at the sensitive area observed at the resonant frequency, only the outside gap responded to glucose solutions of different concentrations. The experimental results of the Reader are shown in Figure 6a. The maximum offset can reach 200 MHz, which reflects the relatively high sensitivity of the Reader. The designed Tag structure was fixed in the middle area of the Reader, and the measurement results are shown in Figure 6b. The sensitivity of the test in direct contact with the Reader appeared low. This may be caused by two reasons. One reason is that after tin is deposited on the surface of the Reader, it hinders the radiation of electromagnetic flux to a certain extent, resulting in transmission loss, causing the frequency change to be insignificant. The second reason is due to the disadvantage of non-contact transmission and the natural non-contact transmission efficiency and sensitivity being lower. Five measurements at each concentration level were taken. Error bars were added to show relative standard deviations (RSD). As for the large scale of the error bars in Figure 6c,d, there may be individual data for the experimental operation tolerance, or DI-water may not be completely discharged from the microfluidic cavity; therefore, a relatively large difference was observed in the measurement results. Fit lines are added to the averages of the measured S_11_ of the bare Reader and RFID system in Figure 6c,d. During the experiment, due to the differences in position, shape, and thickness of the glucose sample, the experimental frequency results could have different deviations; therefore, due to these interference factors, 3.840 GHz was measured for the bare Reader, and such a value falls into the error bars of 100 and 200 mg/dL. In order to eliminate the influence of the abovementioned factors, we use a microfluidic cavity to fix the position, shape, and thickness of the glucose sample. However, due to a few shortcomings of our microfluidic cavity design, there are still some issues remaining to be solved that cause similar problems as the bare Reader did, which are explained as follows: the microfluidic cavity was designed with a square structure, and when the glucose solution was injected, the square structure is difficult to completely fill before the solution is discharged, which will cause a certain tolerance in the experimental results. Besides, in the measurement process, there will be solution residuals remaining in the microfluidic cavity after being rinsed with deionized water several times. It is difficult to completely remove the residual liquid when the gas is passed in such a square cavity. As a result, 3.840 GHz falls into the error bars of 100 and 200 mg/dL in Figure 6d as well. Compared with the results of a single CSRR, although the sensitivity of the biosensor after the composing RFID chip decreased, which may be caused by non-contact reasons, its linearity was greatly improved from R^2^ = 0.88471 to R^2^ = 0.97185 at the same time. The data reveals an absolute sensitivity to glucose concentration levels of 0.31 MHz/mg·dL^−1^ and 10.27 kHz/mg·dL^−1^, the minimum detection limits of 13.79 mg/dL and 1.19 mg/dL, and a response time of less than 1 s, separately. Less than 1 s is required to finish the microwave signal sweep between 3.5 GHz to 4.5 GHz. A comparative analysis based on microwave biosensors is summarized in Table 1, where the detection method and some features of this work are superior to those achieved in previous studies. For example, the proposed design uses a multi-layer structure to achieve a non-contact detection of glucose solution. Although its sensitivity is relatively low compared with the method of direct contact with the detected solution, the sensitivity is relatively good in the non-contact detection method. Besides, the design uses a microfluidic cavity, which can achieve quantitative detection of the solution, reflecting its higher detection accuracy compared to other non-quantitative detection methods.

## 4. Conclusions

This paper presents a non-contact inspired microwave resonator based on the RFID concept integrated with a microfluidic system developed for the sensing of glucose solutions. The overall system is comprised of a Reader, Tag, and microchannel, functioning for healthcare applications such as the detection of the blood level in the human body, etc. Our work provides an efficient solution for the non-contact or possibly short distance signal transmission and biomarker detection method and a feasible fabrication plan for the microwave sensor combined with metal-on-glass, PCB, and PDMS microfluidic channel has been made. The simulation and measurement results show its ability to sense changes in glucose concentrations around the sensitive area. The CSRR Reader can converge electromagnetic flux into the middle gap, and the condensed energy can penetrate the glass substrate to reach the special-customized SSC Tag, as well as the glucose solution. Glucose samples enter the microfluidic cavity through the microchannel and directly contact the Tag, causing the change of the SSC and, thus, changing its resonant frequency. The biosensing response was obtained based on the relationship between glucose concentration and resonant frequency. In general, the proposed microwave design based on the one-to-one correspondence with RFID function will greatly improve the detection speed and efficiency in the face of the complex and huge amount of data that may exist in the future, most importantly, providing an idea for short-distance non-intrusive testing, and some preparations and pavements for future in vitro experiments have been made.

## Figures and Tables

**Table 1:** Performance comparison with other previously reported methods.

Ref.	Sensor structure	Sensitivity	Operating Frequency	Non-Contact	Quantitative Test
[18]	Triple-pole CSRR	0.062 dB/mg·dL^−1^	2.3 GHz	No	Pipette required (600 μL)
[24]	IDC and spiral inductor	1.99 MHz/mg·dL^−1^	2.45 GHz	No	Pipette required (5 μL)
[25]	Air bridge structure	1.08 MHz/mg·dL^−1^	9.20 GHz	No	Pipette required (1 μL)
[26]	CSRR	5 kHz/mg·dL^−1^	2.48 GHz	No	Channel required (0.637 mL)
[27]	Cylindrical dielectric resonator antenna sensor	2.81 kHz/mg·dL^−1^	5.25 GHz	Yes	No
**This work**	**CSRR and SSC**	**10.27 kHz/mg·dL^−1^**	**3.77 GHz**	**Yes**	**Yes** **(1.56 μL)**

## Data Availability

Not applicable.
